# Nauheim Treatment in London

**Published:** 1908-06-27

**Authors:** 


### NAUHEIM TREATMENT IN LONDON.

The various baths, medicinal waters, and system of resistance movements which comprise the elements of what has come to be known as the Nauheim treatment, are widely known and resorted to by the public. There are, in fact, a good number of fairly well-to-do people who habitually resort to Bad-Nauheim, much as others do to Harrogate or Homburg, sometimes with, but often without, the advice or knowledge of their ordinary medical attendant. From them the Hessian Government and the local doctors at Nauheim draw a considerable revenue, for the cure there is expensive as well as prolonged. For those, then, of less affluent circumstances or less leisure it is of some moment to be made aware that in every particular they can obtain in London the advantages of a visit to Nauheim. Not only is the considerable expense of travel and foreign hotel bills saved, but for those whose health is seriously impaired the mere avoidance of the long journey is itself an asset of importance. Moreover, the course of treatment is reasonably moderate in price, and includes the services of two physicians permanently on the staff, who are glad to meet the patient's own medical attendant without extra charge. By them the exact temperature, duration, aeration, and composition of each bath is determined and entered on the medical sheet. The St. George's Nauheim Institution, which offers these facilities to the public is the sole British agency for the Nauheim table and aperient waters. Its premises are at 6 and 7 George Street, Hanover Square, and it is perhaps needless to add that it has no connection with St. George's or any other hospital. The baths are well fitted throughout, and the entire accommodation is designed and carried out in a most successful manner for the purpose in view. Besides the actual baths and dressing-rooms, there are two floors (one for each sex), on which the ordinary appliances of a first-rate gymnasium are provided for those who are strong enough to use them without detriment. Here, too, remedial and corrective exercises for such conditions as spinal curvature and the like can be undertaken under medical supervision. The managers of the Institution have been well advised to reduce the list of complaints for which the Nauheim cure is recommended by the Society of Physicians at Bad-Nauheim; the perfectly reasonable nature of the claims advanced in their prospectus is quite sufficient to differentiate the character of the Institution from the various quack and unqualified exploiters of the invalid public. The equipment and organisation of the Institution we can vouch for as excellent, and the *bona fides* of the management is guaranteed not merely by the very thorough supervision of two staff physicians, but also by the names and standing of those who have sent their patients to George Street for the course of treatment there supplied. The attention of the medical profession may therefore be directed to this means of securing Nauheim treatment without leaving this country.

